# Biomarkers of Toxicant Exposure among Youth in Canada, England, and the United States Who Vape and/or Smoke Tobacco or Do Neither

**DOI:** 10.1158/1055-9965.EPI-24-1338

**Published:** 2025-02-24

**Authors:** David Hammond, Jessica L. Reid, Maciej L. Goniewicz, Ann McNeill, Richard J. O’Connor, Danielle Corsetti, Leonie S. Brose, Bradley Schurr, Deborah Robson

**Affiliations:** 1 School of Public Health Sciences, University of Waterloo, Waterloo, Canada.; 2 Department of Health Behavior, Roswell Park Comprehensive Cancer Center, Buffalo, New York.; 3 Department of Addictions, Institute of Psychiatry, Psychology & Neuroscience, King’s College London, London, United Kingdom.

## Abstract

**Background:**

Few studies examine biomarkers of exposure to vaping and tobacco products among youth. We compared biomarkers for toxicants between youth who vape, smoke, “dual-use”, or neither.

**Methods:**

Participants ages 16 to 19 years in Canada, England, and the United States completed surveys and self-collected urine samples between September 2019 and January 2022 (*N* = 364). Urine was tested for metabolites of tobacco-specific nitrosamine NNK (4-(methylnitrosamino)-1-(3-pyridyl)-1-butanone) and six volatile organic compounds. Regression models examined differences in biomarker concentrations by past-week tobacco smoking and vaping, adjusting for creatinine, age, sex, country, and cannabis use.

**Results:**

Compared with no vaping/smoking, exclusive vaping was associated with similar exposure to acrolein and acrylonitrile but higher exposure to toluene (*P* = 0.04) and acrylamide (*P* = 0.034, only in sensitivity analysis using past 24-hour measure). Compared with dual use or exclusive smoking, exclusive vaping was associated with lower exposure to NNK, acrolein, acrylamide, and acrylonitrile (*P* ≤ 0.01) but higher toluene exposure than dual use (*P* = 0.012). Exposure was similar for dual-use and exclusive smoking. Benzene and xylene biomarkers were detected in <5% and not compared. Among those smoking, NNK exposure was higher in the United States (geometric mean = 25.4 pg/mg creatinine) versus Canada (16.1 pg/mg; *P* = 0.006) and England (14.1 pg/mg; *P* = 0.018).

**Conclusions:**

Youth exclusively vaping had similar exposure as no vaping/smoking except for two volatile organic compounds and lower exposure than smoking or dual use except toluene. Higher NNK levels among US youth who smoke likely reflect differences in tobacco blend.

**Impact:**

Findings are generally consistent with literature indicating lower toxicant exposure from vaping versus smoking but elevated exposure versus no use for some.

## Introduction

Vaping e-cigarettes has emerged as a popular mode of nicotine delivery among young people (1). Vaping can pose some level of health risk; however, there is a high degree of uncertainty about the magnitude of risk, particularly among those who have never smoked (2, 3). Vaping aerosol includes a range of chemicals and ultrafine particles known to cause detrimental health effects (3). These include metals and volatile organic compounds (VOC), which are important respiratory and cardiovascular toxicants (4). VOCs can form from thermal degradation of constituents in e-liquids, including widely used solvents (2, 3, 5). Previous studies have also detected tobacco-specific nitrosamines (TSNA) in some vaping liquids/aerosols (6, 7), which are a notable class of carcinogenic compounds that naturally occur in tobacco and increase during the curing process (3, 8). The presence of TSNAs in e-liquids is avoidable and may reflect impurities and deficiencies in manufacturing practices (e.g., contamination from nicotine extracted from tobacco). With the notable exception of nicotine and some metals, vaping aerosol contains fewer numbers and lower levels of toxicants than tobacco smoke (9, 10).

As of yet, there is no available evidence whether vaping causes chronic diseases, including respiratory disease, cardiovascular disease, or cancer; these diseases have a delayed onset and additional epidemiologic data over a longer time will be required to examine the impact of vaping on chronic disease (3, 11, 12). However, biological plausibility has been established by experimental and preclinical studies demonstrating the effects of vaping aerosol on oxidative stress, inflammation, endothelial function, and platelet activation (3, 11).

In the absence of long-term epidemiologic data, biomarkers can serve as intermediate indicators and potential early warning signals of disease (13). Several studies have investigated biomarkers of exposure (BoE), which measure the levels of toxicants or their metabolites in biological fluids such as urine, blood, saliva, and exhaled-breath (13). Commonly used biomarkers of exposure to tobacco-related toxicants have been correlated with an increased risk of major types of cancer, respiratory, and cardiovascular diseases (14). An increasing number of studies have also examined biomarkers of potential harm, which assess biological changes in the body as a result of exposure, such as oxidative stress, inflammation, and endothelial function (14).

To date, virtually all vaping biomarker studies have been conducted among adults who had smoked and switched to e-cigarettes (2, 9). Reviews and meta-analyses of BoE studies demonstrate reduced levels of exposure to most toxicants among adults who previously smoked and switched to exclusively vaping, with notable exceptions for nicotine and some metals and inconclusive findings for some VOCs, such as formaldehyde and toluene (2, 3, 9, 14–16).

Fewer biomarker studies have compared vaping to “no use” (i.e., neither vaping nor smoking). The evidence to date suggests that, compared with adults that neither smoke nor vape, adults who vape have higher levels of exposure to some TSNAs, VOCs (e.g., acrylonitrile), metals (e.g., cadmium and selenium), and propylene glycol compared with nonusers, with mixed findings for other BoEs (e.g., acrolein, benzene, and chromium; refs. 2, 9, 14, 16). A major limitation of this literature is that virtually all research on BoEs from “exclusive vaping” has been conducted with adults who formerly smoked, often vaping for a relatively brief period (17). Thus, most studies that compare “exclusive vaping” with “no use” are confounded by the smoking history of those “exclusively vaping”, given that half-lives of biomarkers vary greatly, from minutes to several years.

To date, there is little evidence among youth on BOEs from tobacco and vaping, including exclusive vaping, or among young people without a smoking history. A study from the United States found no difference in 4-(methylnitrosamino)-1-(3-pyridyl)-1-butanol (NNAL; one of the biomarkers for TSNAs) levels between youth who used e-cigarettes and those who smoked tobacco or did not use either. However, the small sample size of youth using e-cigarettes (*n* = 12) provided limited power (18). Another US study reported higher levels of metabolites for several VOCs (specifically acrylonitrile, acrolein, propylene oxide, acrylamide, and crotonaldehyde) among youth who vaped in the last 24 hours versus nonusing controls (19).

BoEs also provide a means of comparing differences between products, including potential differences in products across countries (14). For example, the tobacco blend in cigarettes differs across countries and has been associated with markedly different levels of TSNAs in cigarettes (20). Biomarker studies conducted with adult smokers indicate substantially higher levels of NNK and other TSNA exposure among smokers using “US blended” cigarettes; however, we are unaware of any studies that have compared TSNA levels among youth from different countries (21, 22). Differences in vaping products across countries have also been observed, in some cases within the same brand (23, 24); however, there are little data on TSNA exposure among young people who vape.

The current study examined biomarkers of exposure to selected toxicants among youth in three countries: Canada, England, and the United States. The study had two primary aims: (1) to examine differences in exposure to toxicants among youth who vape, smoke tobacco, both vape and smoke (“dual use”), or do neither (“no use”), and (2) to examine country-level differences in exposure levels. For aim 1, we hypothesized that (1) concentrations of TSNA and VOC biomarkers would be higher among those who smoke tobacco (exclusive or dual use) versus those who do not (no use and exclusive vaping), with no difference between those who exclusively smoke and those who dual use, and (2) concentrations of VOC biomarkers would be higher among those who vape (exclusive or dual use) than those reporting no use, with no differences for the TSNA biomarker. For aim 2, we hypothesized that biomarker concentrations would vary by country; among those who smoke (exclusive or dual use), concentrations of the TSNA biomarker would be higher among those in the United States than in England and Canada because of tobacco blend.

## Materials and Methods

### Participants

The current study was an extension of the ITC Youth Tobacco and Vaping Surveys, online surveys conducted with national samples of youth ages 16 to 19 years in Canada, England, and the United States (see https://davidhammond.ca/projects/e-cigarettes/itc-youth-tobacco-ecig/, Technical Reports for Waves 3 through 5; ref. 25). After completing online surveys, participants ages 16 to 19 years (both males and females) were recruited from commercial panels in each country (see Supplementary Fig. S1 for the participation flowchart). Eligibility was based on categorization of smoking and vaping behavior (determined by responses to survey items) into one of four groups [past-week vaping, past-week cigarette smoking, past-week vaping and smoking, or no use (no smoking, vaping, or cannabis use in the past 30 days)]; participants were not eligible if they had failed a quality check question asking for the current month or if they belonged to a panel which opted out of inviting participants to this additional study (see Technical Report). Initial sample size targets of 180 participants across countries for each user group (total *n* = 720) were based on power calculations for individual biomarkers; for example, sample sizes of 180 per user group were estimated to provide 80% power to detect pairwise differences of 85 ng/g creatine for 3-hydroxypropyl mercapturic acid (3HPMA), the biomarker for acrolein (assuming a control mean = 327.8 ng/g and SD = 303.5 ng/g), for a two-tailed test in which α = 0.05.

All participants were provided with study information and asked to indicate their consent by selecting an option in the online survey; in addition, parental consent was ascertained for participants younger than 18 years of age via the same method. Participants received 50CAD/40USD/£30 remuneration via Amazon gift card (or, in Canada only, choice of Amazon gift card or Interac payment) sent by email. This study was reviewed and received ethics clearance through a University of Waterloo Research Ethics Committee (ORE#21847/31017), King’s College London Psychiatry, Nursing & Midwifery Research Ethics Subcommittee (#13748), and a Roswell Park Comprehensive Cancer Center Ethics Committee (STUDY00001085/P-522019). This study was conducted in accordance with the principles of the Declaration of Helsinki.

### Sample collection

Sample collection occurred between September 2019 and January 2022 (17 in 2019, 257 in 2020, 89 in 2021, and one in 2022). Participants were sent a urine collection kit by courier, which included instructions and materials required for self-collection and sample return, as well as a one page paper-and-pencil questionnaire. Participants were asked to collect their first urine after waking, fill two sample tubes (up to 40 mL), and package them with a frozen gel pack in a styrofoam box and shipping box (supplied). The samples and questionnaires were sent by courier (priority service) back to the University of Waterloo for participants in Canada, to Roswell Park Comprehensive Cancer Center for participants in the United States, or by first-class mail (1–2 days) to the National Institute for Health Research BioResource Center Maudsley (at King’s College London) for participants in England. Samples were immediately placed in a −20°C freezer for storage. Samples in England were centrifuged within 7 days of receipt to remove any cellular material, per local regulations. At the completion of data collection, samples in Canada and England were shipped to Roswell Park Comprehensive Cancer Center on dry ice for storage and testing. The methodology used to collect and ship the samples has been previously established (26).

### Survey measures and vaping/smoking status

On the questionnaire completed at the time of sample collection, participants self-reported last use (less than 1 hour ago; 1–6 hours ago; 7–12 hours ago; 12–24 hours ago; 1–7 days ago; or not at all in last 7 days) of each of the following: “Used an e-cigarette/vaped,” “Smoked a regular cigarette,” “Smoked any other tobacco (cigar, cigarillo, bidi, shisha, etc.),” “Smoked cannabis/marijuana,” “Vaped cannabis/marijuana,” “Used smokeless tobacco (chew, pinch, snuff, snus),” “Used nicotine replacement therapy (patches, gum, lozenges, etc.) or nicotine pouches,” “Ate grilled meat (i.e., cooked over flame or charcoal, or with black grill marks)", and [from Wave 4 (August 2020) onward] “Were in the presence of someone smoking cigarettes or tobacco inside (home, car, etc.)”. See supplementary Materials and Methods for questionnaires.

Participants were classified into four categories based on self-reported past 7-day vaping and tobacco smoking (including cigarettes and/or other tobacco products) at the time of sample collection: no use (neither vaped nor smoked), exclusive vaping (vaped but did not smoke), exclusive smoking (smoked but did not vape), or dual use (both vaped and smoked). As classifications were based on behavior, participants could not be randomly assigned to groups.

### Biomarker testing

Urine samples were tested at the Nicotine and Tobacco Product Assessment Resource lab at Roswell Park Comprehensive Cancer Center. Samples were identified by a code to ensure lab personnel were blinded to the smoking/vaping status of participants. Samples were tested for metabolites of a TSNA (NNAL, a metabolite of NNK) and six VOCs [acrolein (3HPMA), acrylamide (2CaHEMA), acrylonitrile (2CyEMA), benzene (PhMA), toluene (BzMA), and xylene (24MPhMA)], as described elsewhere (27–29). Biomarkers were selected to examine a toxic constituent in tobacco products that has previously been identified in some vaping products (NNAL), and VOC metabolites, which have been identified as a concern for vaping products and for which there is little existing evidence (9). Biomarker concentrations were normalized for creatinine, to adjust for differences in hydration status on sample collection. Metabolites of interest, including units, testing limits, and relevance of parent compounds to health, are summarized in Table 1.

**Table 1.** Toxicants of interest and their biomarkers measured in the study, including analyte testing limits.

Testing group	Parent compound	Classification/clinical relevance	Metabolite	Common name (47)	Unit	LLOQ (ng/mL)
TSNA	4-(methylnitrosamino)-1-(3-pyridyl)-1-butanone (NNK)	Known human carcinogen (IARC group 1; ref. 48)	4-(methylnitrosamino)-1-(3-pyridyl)-1-butanol	NNAL	pg/mL	3.0
VOCs	Acrolein	Probable carcinogen (IARC group 2A; ref. 38), respiratory toxicant, and cardiovascular toxicant (FDA HPHC; ref. 49)	3-hydroxypropyl mercapturic acid	3HPMA	ng/mL	30.0
	Acrylamide	Probable carcinogen (IARC group 2A; ref. 38) and neurotoxic (27)	2-carbamoyl-2-hydroxy-ethyl mercapturic acid	2CaHEMA	ng/mL	10.0
	Acrylonitrile	Possible carcinogen (IARC group 2B; ref. 38) and respiratory toxicant (FDA HPHC; ref. 39)	2-cyanoethyl mercapturic acid	2CyEMA	ng/mL	5.0
	Benzene	Known carcinogen (IARC group 1; ref. 38), cardiovascular toxicant, and reproductive or developmental toxicant (FDA HPHC; ref. 39)	Phenyl mercapturic acid	PhMA	ng/mL	2.0
	Toluene	Respiratory toxicant and reproductive or developmental toxicant (FDA HPHC; ref. 39)	Benzyl mercapturic acid	BzMA	ng/mL	2.0
	Xylene	Not classified by IARC (group 3); neurologic effects (50)	2,4-dimethylphenyl mercapturic acid	24MPhMA	ng/mL	2.0
Control	Creatine	Indicator of urine dilution	Creatinine		mg/dL	Reference range 1.2–346.5

Abbreviations: HPHC, harmful or potentially harmful constituent; IARC, International Agency for Research on Cancer; LLOQ, lowest limit of quantitation.

### Statistical analysis

Demographic characteristics of the four categories of past 7-day vaping and tobacco smoking were compared using χ^2^ tests for sex and country and linear regression for age.

Biomarker values below the assay limit of quantitation (LOQ) were imputed using the common substitution formula LOQ/√2. All biomarker values were normalized for creatinine concentration, calculated by dividing the biomarker concentration in urine by creatinine concentration in urine (expressed as mg/mL). Data points from participants with creatinine level values outside of the reference range (30) were excluded (*n* = 3; *n* = 1 ≤ 10 mg/dL, and *n* = 2 > 370 mg/dL). Extreme values exceeding three SDs from the mean were excluded from analysis on a case-wise basis. For each biomarker, the number of participants with a value above the LOQ and the geometric mean concentration are reported, among each vaping and tobacco smoking status group, overall, and by country. Biomarkers with >95% of values below the LOQ were not analyzed further. Statistical analysis was conducted using IBM SPSS Statistics, version 29 (IBM Corporation, 2023; RRID: SCR_002865).

Analyses were preregistered using the Open Science Framework system (https://osf.io/9z3cu) and were as follows for each specific aim.

#### Aim 1

Separate linear regression models were conducted for each biomarker (using log-transformed values) to examine differences based on smoking and vaping status in the past 7 days (no use, exclusive vaping, exclusive smoking, and dual use; pairwise comparisons between each group). Models were adjusted for creatinine, age, sex, country, and cannabis use in the past 7 days (no use, exclusive vaping, exclusive smoking, and both vaping and smoking cannabis).

#### Aim 2

To examine country differences, a model was estimated for TSNA (using log-transformed values) and adjusted for creatinine, age, sex, country, and cannabis use in the past 7 days, which included an interaction term between past-week vaping/smoking status and country and specified contrasts comparing countries (pairwise) among all those who smoked in the past week (exclusive smoking or dual use).

### Sensitivity analysis

Two different sensitivity analyses were preregistered. For the first, the aim 1 models described above for all biomarkers were conducted using smoking and vaping status in the past 24 hours (no use, exclusive vaping, exclusive smoking, and dual use), which is a more stringent measure of recent use (vs. past-week use). Second, the models described above for all biomarkers were conducted adjusting concurrently for any past-week use of nicotine replacement therapy, any past-week use of smokeless tobacco products, and any past-week exposure to second-hand smoke (by adding variables for each to the models). Further to the preregistration, the model for acrylamide exposure (2CaHEMA concentration) was estimated adjusting for eating grilled meat in the past 7 days [which may increase exposure to acrylamide (31)]. Finally, the aim 1 models using past-week and past 24-hour use variables were conducted using biochemical thresholds to supplement self-reported smoking and vaping status. Participants in the “no use” group with urinary cotinine concentration above 50 ng/mg creatinine were excluded (per established guidelines; ref. 32), as were participants in the vaping, smoking, and dual-use groups in which the presence of cotinine was not detected (i.e., below the lowest LOQ of 5 ng/mL). A complete analysis of cotinine values is reported elsewhere (33).

### Data availability

The data generated in this study are available upon reasonable request from the corresponding author to researchers who submit a proposal that is approved by the principal investigator.

## Results

### Sample

Characteristics of the 364 participants ages 16 to 19 years who provided a usable sample and completed the questionnaire are shown in Table 2, by past-week smoking and vaping categories. There were no differences between groups in age (Wald χ^2^ = 0.05, *P* = 0.82) or sex (Pearson χ^2^ = 6.4, *P* = 0.09), although the country distribution varied significantly (Pearson χ^2^ = 16.3, *P* = 0.01). Supplementary Table S1 presents characteristics and past-week behaviors/exposures by country.

**Table 2.** Participant characteristics and past-week behaviors and exposures at the time of sample collection, by past-week vaping and tobacco smoking group (*n* = 364).

	No use	Vaping (exclusive)	Smoking^a^ (exclusive)	Dual use	Total
	*n* = 146	*n* = 73	*n* = 68	*n* = 77	*N* = 364
	*n* (%)	*n* (%)	*n* (%)	*n* (%)	*n* (%)
Age (mean, SD)	17.49 (1.05)	17.78 (1.08)	17.51 (1.02)	17.47 (1.18)	17.55 (1.08)
Sex					
Male	63 (43.2%)	25 (34.2%)	31 (45.6%)	42 (54.5%)	161 (44.2%)
Female	83 (56.8%)	48 (65.8%)	37 (54.4%)	35 (45.5%)	203 (55.8%)
Country					
Canada	52 (35.6%)	35 (47.9%)	16 (23.5%)	26 (33.8%)	129 (35.4%)
England	57 (39.0%)	14 (19.2%)	33 (48.5%)	27 (35.1%)	131 (36.0%)
United States	37 (25.3%)	24 (32.9%)	19 (27.9%)	24 (31.2%)	104 (28.6%)
Past-week cigarette smoking					
No	146 (100%)	73 (100%)	4 (5.9%)	4 (5.2%)	227 (62.4%)
Yes	0 (0%)	0 (0%)	64 (94.1%)	73 (94.8%)	137 (37.6%)
Past-week other tobacco smoking					
No	146 (100%)	72 (98.6%)	53 (77.9%)	57 (74.0%)	328 (90.9%)
Yes	0 (0%)	0 (0%)	13 (19.1%)	20 (26.0%)	33 (9.1%)
Missing	0 (0%)	1 (1.4%)	2 (2.9%)	0 (0%)	3 (0.8%)
Past-week smokeless tobacco					
No	146 (100%)	73 (100%)	66 (97.1%)	72 (93.5%)	357 (98.1%)
Yes	0 (0%)	0 (0%)	2 (2.9%)	5 (6.5%)	7 (1.9%)
Past-week NRT use					
No	145 (99.3%)	71 (97.3%)	65 (95.6%)	70 (909%)	351 (96.4%)
Yes	1 (0.7%)	2 (2.7%)	3 (4.4%)	6 (7.8%)	12 (3.3%)
Missing	0 (0%)	0 (0%)	0 (0%)	1 (1.3%)	1 (0.3%)
Past-week SHS exposure^b^					
No	61 (41.8%)	31 (42.5%)	14 (20.6%)	14 (18.2%)	120 (33.0%)
Yes	24 (16.4%)	23 (31.5%)	27 (39.7%)	41 (53.2%)	115 (31.6%)
Missing	61 (41.8%)	19 (26.0%)	27 (39.7%)	22 (28.6%)	129 (35.4%)
Past-week cannabis smoking					
No	140 (95.9%)	44 (60.3%)	48 (70.6%)	35 (45.5%)	267 (73.4%)
Yes	6 (4.1%)	29 (39.7%)	20 (29.4%)	39 (50.6%)	94 (35.8%)
Missing	0 (0%)	0 (0%)	0 (0%)	3 (3.9%)	3 (0.8%)
Past-week cannabis vaping					
No	144 (98.6%)	61 (83.6%)	65 (95.6%)	61 (79.2%)	331 (90.9%)
Yes	2 (1.4%)	12 (16.4%)	3 (4.4%)	16 (20.8%)	33 (9.1%)

Abbreviations: NRT, nicotine replacement therapy; SHS, secondhand smoke.

a Includes cigarettes and other smoked tobacco (cigar, cigarillo, bidi, shisha, etc.).

b Question about SHS added at Wave 4 (2020_Aug_).

### BoE

Table 3 shows the number of samples with concentration of biomarkers above the LOQ within each smoking/vaping status group, as well as the geometric mean concentrations of each, normalized for creatinine. More than 95% of samples in each user group had levels below the LOQ for benzene (PhMA) and xylene (24MPhMA); hence, concentrations are not reported and groups were not compared in regression models.

**Table 3.** Biomarkers of exposure within past-week vaping and tobacco smoking status groups, *n* (%) samples with concentration above LLOQ and geometric mean (SD) concentrations, normalized for mg creatinine.

	TSNA	VOC biomarkers					
	NNK (NNAL)	Acrolein (3HPMA)	Acrylamide (2CaHEMA)	Acrylonitrile (2CyEMA)	Benzene (PhMA)	Toluene (BzMA)	Xylene (24MPhMA)
LLOQ	3.0 pg/mL	30.0 ng/mL	10.0 ng/mL	5.0 ng/mL	2.0 ng/mL	2.0 ng/mL	2.0 ng/mL
**Presence**, *n* present/total (%)							
No use	26/146 (17.8%)	144/146 (98.6%)	127/146 (87.0%)	62/146 (42.5%)	0/146 (0%)	132/146 (90.4%)	0/146 (0%)
Past-week vaping	14/73 (19.2%)	73/73 (100%)	71/73 (97.3%)	49/73 (67.1%)	1/73 (1.4%)	68/73 (93.2%)	0/73 (0%)
Past-week smoking	52/68 (76.5%)	68/68 (100%)	64/68 (94.1%)	58/68 (85.3%)	2/68 (2.9%)	61/68 (89.7%)	0/68 (0%)
Dual use (past-week vaping and smoking)	54/77 (70.1%)	76/77 (98.7%)	69/77 (89.6%)	62/77 (80.5%)	3/77 (3.9%)	60/77 (77.9%)	0/77 (0%)
**Concentration**,^a^ geometric mean (SD)	pg/mg	ng/mg	ng/mg	ng/mg	ng/mg	ng/mg	ng/mg
No use	2.1 (2.6)	327.8 (303.8)	19.2 (11.5)	3.5 (4.95)	n/a^b^	3.97 (4.10)	n/a^b^
Past-week vaping	1.8 (4.4)	360.7 (310.3)	23.3 (15.2)	4.7 (35.9)	n/a^b^	4.48 (10.10)	n/a^b^
Past-week smoking	20.4 (89.9)	633.6 (1,253.9)	35.6 (31.9)	31.0 (97.1)	n/a^b^	4.67 (3.91)	n/a^b^
Dual use (past-week vaping and smoking)	15.1 (77.5)	605.0 (1,303.1)	35.0 (141.0)	24.1 (82.9)	n/a^b^	3.90 (3.97)	n/a^b^

Abbreviation: n/a, not applicable.

a Estimates of concentration exclude outliers (*n* = 4 for 3HPMA, *n* = 7 for 2CaHEMA, *n* = 6 for 2CyEMA, *n* = 8 for BzMA, and *n* = 6 for NNAL), participants with creatinine values outside of the reference range (*n* = 3), and samples in which the sample matrix affected accurate detection of results (*n* = 1 for 3HPMA, *n* = 15 for 2CaHEMA, *n* = 1 for 2CyEMA, *n* = 1 for BzMA, and *n* = 3 for NNAL). For NNAL, *n* = 1 value <LLOQ cutoff but quantified was included.

b >95% of samples had levels below the LOQ.

### Differences in TSNA and VOC biomarkers by past-week vaping and smoking

**Figure 1.**
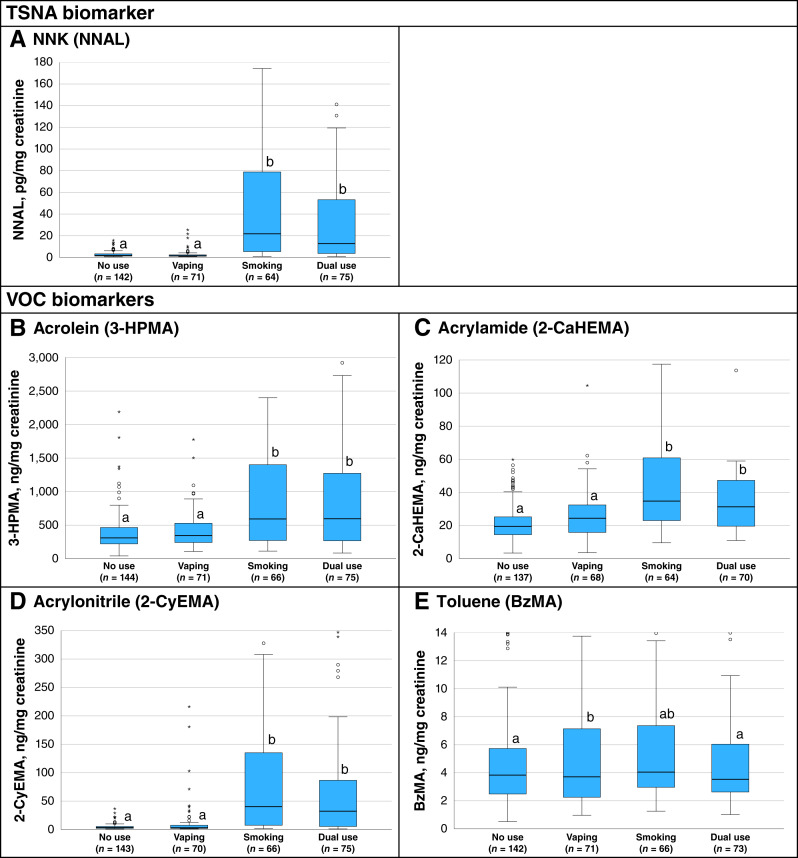
Biomarkers of exposure to TSNA and VOCs by past-week vaping and tobacco smoking status. Boxplots show median (IQR) creatinine-adjusted concentrations of biomarkers for (**A**) NNK (NNAL), (**B**) acrolein (3HPMA), (**C**) acrylamide (2CaHEMA), (**D**) acrylonitrile (2CyEMA), and (**E**) toluene (BzMA), within groups based on past-week vaping and smoking; whiskers depict minimum and maximum non-outlier values (some outliers excluded for clarity of presentation). Different letters denote significant differences between groups (at *P* < 0.05), in pairwise comparisons from separate linear regression models for each biomarker, adjusting for creatinine, country, age, sex, and past-week cannabis use (e.g., in **A**, the “no use” and “vaping” groups are both marked “a”, indicating that they do not differ significantly from one another, but they differ significantly from the “smoking” and “dual use” groups that are both marked “b”).

Figure 1 shows box and whisker plots for five biomarkers of exposure (NNAL, 3HPMA, 2CaHEMA, 2CyEMA, and BzMA), by past-week vaping and tobacco smoking status, and indicates significant (at *P* < 0.05) differences between groups. Differences by past-week vaping and smoking status groups (indicated in Fig. 1 by different letters) were observed for each of the five biomarkers tested (NNAL, 3HPMA, 2CaHEMA, 2CyEMA, and BzMA), adjusting for age, sex, country, past-week cannabis use, and creatinine concentration. Concentrations of biomarkers for exposure to NNK (NNAL), acrolein (3HPMA), acrylamide (2CaHEMA), and acrylonitrile (2CyEMA) were significantly (*P* ≤ 0.01) lower among those who exclusively vaped than among those who exclusively smoked or dual used; there were no significant differences between those who exclusively vaped and those who did not use or between those who exclusively smoked and those who dual used. Exposure to toluene (BzMA) among those who exclusively vaped was higher than among those who did not use (*P* = 0.039) or dual used (*P* = 0.012). See Table 4 for model estimates for all contrasts.

**Table 4.** Comparisons between past-week vaping and tobacco smoking status groups^a^ for biomarkers of exposure, ng/mg.

	Vaping/smoking status	Vaped vs. no use	Smoked vs. no use	Dual use vs. no use	Vaped vs. smoked	Vaped vs. dual use	Smoked vs. dual use
	Model effect	B (95% CI; *P* value) for comparison					
NNAL^b^ (*n* = 351)	Wald χ^2^ = 194.49 (*P* < 0.001)	−0.10 (−0.44–0.24; *P* = 0.56)	**1.99 (1.64–2.34; *P* < 0.001)**	**1.67 (1.32–2.03; *P* < 0.001)**	−**2.09 (**−**2.49–1.69; *P* < 0.001)**	−**1.77 (**−**2.15–1.32; *P* < 0.001)**	0.31 (−0.07–0.70; *P* = 0.11)
3HPMA (*n* = 355)	Wald χ^2^ = 28.79 (*P* < 0.001)	0.11 (−0.15–0.37; *P* = 0.39)	**0.59 (0.33–0.86; *P* < 0.001)**	**0.57 (0.30–0.84; *P* < 0.001)**	−**0.48 (**−**0.78**–**0.18; *P* = 0.002)**	−**0.45 (**−**0.74**–**0.16; *P* = 0.002)**	0.03 (−0.27–0.32; *P* = 0.86)
2CaHEMA (*n* = 338)	Wald χ^2^ = 31.64 (*P* < 0.001)	0.14 (−0.05–0.33; *P* = 0.15)	**0.48 (0.29–0.67; *P* < 0.001)**	**0.42 (0.22–0.61; *P* < 0.001)**	−**0.34 (**−**0.56**–**0.13; *P* = 0.002)**	−**0.28 (**−**0.49**–**0.06; *P* = 0.011)**	0.68 (−0.15–0.28; *P* = 0.53)
2CyEMA (*n* = 353)	Wald χ^2^ = 129.86 (*P* < 0.001)	0.20 (−0.15–0.55; *P* = 0.25)	**1.78 (1.43–2.13; *P* < 0.001)**	**1.42 (1.06–1.78; *P* < 0.001)**	−**1.58 (**−**1.98–1.17; *P* < 0.001)**	−**1.22 (**−**1.61–0.83; *P* < 0.001)**	0.36 (−0.04–0.75; *P* = 0.075)
BzMA (*n* = 351)	Wald χ^2^ = 7.06 (*P* = 0.07)	**0.23 (0.01–0.44; *P* = 0.039)**	0.03 (−0.19–0.25; *P* = 0.78)	−0.08 (−0.30–0.14; *P* = 0.49)	0.20 (−0.05–0.44; *P* = 0.12)	**0.31 (0.07–0.54; *P* = 0.012)**	0.11 (−0.13–0.35; *P* = 0.37)

Abbreviation: CI, confidence interval.

Bolded values indicate statistical significance at the *P* < 0.05 level.

a From separate linear regression models for each biomarker (using log-transformed values) adjusted for creatinine, age, sex, country, and cannabis use in the past 7 days (no use, exclusive vaping, exclusive smoking, and both vaping and smoking).

b pg/mg creatinine.

### Differences in TSNA biomarker by country

Table 5 shows the geometric mean concentrations of each biomarker metabolite, normalized for creatinine, within each smoking/vaping status group and by country. Adjusting for age, sex, country, past-week smoking/vaping status, past-week cannabis use, and creatinine concentration, NNAL concentrations were significantly higher among those who had smoked tobacco in the past week (exclusive or dual; Canada *n* = 42, England *n* = 57, and United States *n* = 40) in the United States (geometric mean = 25.4 pg/mg creatinine, SD = 106.1) compared with Canada (16.1 pg/mg creatinine, SD = 63.0; B = −0.72, *P* = 0.006) and England (14.1 pg/mg creatinine, SD = 74.2; B = −0.57, *P* = 0.018); there was no evidence for a difference between Canada and England (B = −0.15, *P* = 0.55).

**Table 5.** Geometric means for biomarkers of exposure within past-week vaping and tobacco smoking status groups by country, mean (SD) concentration, ng/mg creatinine.

	TSNA	VOC biomarkers					
	NNK (NNAL)^a^	Acrolein (3HPMA)	Acrylamide (2CaHEMA)	Acrylonitrile (2CyEMA)	Benzene (PhMA)	Toluene (BzMA)	Xylene (24MPhMA)
**Canada**	*n* = 127	*n* = 126	*n* = 116	*n* = 126	*n* = 125	*n* = 124	*n* = 124
No use (*n* = 52)	2.0 (2.2)	344.3 (322.6)	18.4 (11.1)	2.8 (5.7)	n/a^b^	3.81 (3. 48)	n/a^b^
Past-week vaping (*n* = 35)	1.6 (1.9)	374.7 (208.9)	24.4 (8.6)	4.7 (39.0)	n/a^b^	3.89 (6.66)	n/a^b^
Past-week smoking (*n* = 16)	16.9 (74.8)	640.6 (885.8)	50.8 (44.8)	25.4 (84.8)	n/a^b^	5.30 (4.28)	n/a^b^
Dual use (*n* = 26)	15.7 (55.5)	710.3 (1,340.0)	59.1 (244.3)	27.4 (97.4)	1.24 (1.47)^c^	4.16 (3.54)	n/a^b^
**England**	*n* = 126	*n* = 129	*n* = 124	*n* = 128	*n* = 127	*n* = 128	*n* = 125
No use (*n* = 57)	2.0 (2.4)	326.2 (196.6)	20.9 (11.8)	4.2 (3.2)	n/a^b^	4.43 (4.49)	n/a^b^
Past-week vaping (*n* = 14)	2.6 (6.6)	289.1 (282.4)	23.0 (12.8)	4.7 (26.5)	n/a^b^	4.11 (13.41)	n/a^b^
Past-week smoking (*n* = 33)	17.3 (94.4)	695.5 (1,545.7)	33.9 (25.8)	34.2 (102.0)	1.27 (0.97)^c^	4.38 (3.13)	n/a^b^
Dual use (*n* = 27)	11.1 (40.3)	531.0 (911.0)	30.6 (31.3)	23.4 (81.9)	n/a^b^	4.00 (3.52)	n/a^b^
**United States**	*n* = 99	*n* = 101	*n* = 99	*n* = 100	*n* = 100	*n* = 100	*n* = 102
No use (*n* = 37)	2.4 (3.3)	301.3 (398.3)	17.9 (11.4)	3.5 (5.8)	n/a^b^	3.61 (4.11)	n/a^b^
Past-week vaping (*n* = 24)	1.8 (5.2)	389.5 (422.3)	22.1 (22.1)	4.6 (37.5)	0.98 (0.53)^c^	5.72 (11.76)	n/a^b^
Past-week smoking (*n* = 19)	31.7 (94. 6)	529.3 (880.9)	28.8 (19.5)	30.7 (101.7)	n/a^b^	4.69 (4.79)	n/a^b^
Dual use (*n* = 24)	21.1 (116.8)	587.5 (1,654.5)	25.1 (11.3)	21.6 (65.7)	1.21 (0.65)^c^	3.45 (5.06)	n/a^b^

Estimates of concentration exclude outliers (*n* = 4 for 3HPMA, *n* = 7 for 2CaHEMA, *n* = 6 for 2CyEMA, *n* = 8 for BzMA, and *n* = 6 for NNAL), participants with creatinine values outside of the reference range (*n* = 3), and samples in which the sample matrix affected accurate detection of results (*n* = 1 for 3HPMA, *n* = 15 for 2CaHEMA, *n* = 1 for 2CyEMA, *n* = 1 for BzMA, and *n* = 3 for NNAL). For NNAL, *n* = 1 value <LLOQ cutoff but quantified was included.

Abbreviations: TSNA, tobacco-specific nitrosamine; VOC, volatile organic compound.

a pg/mg creatinine.

b 100% of samples had levels below the LOQ.

c >95% of samples had levels below the LOQ. For benzene, *n* = 6 with levels above LOQ (*n* = 1 dual use in Canada, *n* = 2 smoking in England, and *n* = 1 vaping and *n* = 2 dual use in the United States).

### Sensitivity analyses

Using smoking and vaping status in the past 24 hours rather than the past 7 days (see Supplementary Table S2 for biomarker presence and concentration estimates) yielded similar results of linear regression models for each biomarker concentration (adjusting for age, sex, country, past-week cannabis use, and creatinine concentration). In general, effect sizes were slightly larger using the past 24-hour measure (see Supplementary Table S3 for model estimates), and a few comparisons were significant only in the models using the 24-hour measure; 2CaHEMA (acrylamide) was higher among those exclusively vaping compared with those not using, and NNAL and 2CyEMA (acrylonitrile) were higher among those exclusively smoking than among those dual using.

Sensitivity analyses were also undertaken that adjusted models concurrently for any past-week use of nicotine replacement therapy, any past-week use of smokeless tobacco products, and any past-week exposure to second-hand smoke (see Table 2 for frequencies by smoking/vaping groups). These variables were not significantly associated with any biomarkers except NNAL, which was associated with smokeless tobacco use (*P* = 0.030) and second-hand smoke exposure (*P* = 0.027). There were few differences in the main effects or pairwise comparisons between user groups, although the effects of user groups were slightly attenuated in most cases.

Further to the preregistration, the model described above for 2CaHEMA was repeated adjusting for eating grilled meat in the past 7 days (which was reported by 41.5% of respondents), but this variable was not significantly associated with concentration (Wald χ^2^ = 1.4, *P* = 0.23), and estimates for between-group comparisons based on past-week smoking and vaping were very similar.

Finally, sensitivity analyses were conducted using biochemical thresholds to verify self-reported smoking and vaping status, which excluded 56 participants (*n* = 3 “no use” with cotinine above 50 ng/mg creatinine; n = 21 vaping, *n* = 19 smoking, and *n* = 15 dual use with cotinine not detected) from the past-week analysis and 38 participants (*n* = 8 “no use” with cotinine above 50 ng; *n* = 12 vaping, *n* = 10 smoking, and *n* = 8 dual use with cotinine not detected) from the past 24-hour analysis. Supplementary Table S4 shows the presence and mean concentrations of biomarkers within each past-week smoking/vaping status group using the cotinine-validated sample. As shown in Supplementary Table S5, the pattern of findings from models using biochemical thresholds was largely consistent with the original analyses. For models examining past-week use, modest differences were observed between the original analysis based on the self-report measure and the model based on the cotinine-validated measure for the contrasts for vaping versus “no use” for 2CyEMA (B_SR (self-report)_ = 0.20, *P* = 0.25 vs. B_BIO (biochemically validated)_ = 0.40, *P* = 0.034) and BzMA (B_SR_ = 0.23, *P* = 0.039 vs. B_BIO_ = 0.23, *P* = 0.077); for vaping versus dual use for BzMA (B_SR_ = 0.31, *P* = 0.012 vs. B_BIO_ = 0.26, *P* = 0.066); and for smoking versus dual use for NNAL (B_SR_ = 0.31, *P* = 0.11 vs. B_BIO_ = 0.48, *P* = 0.014) and 2CyEMA (B_SR_ = 0.36, *P* = 0.075 vs. B_BIO_ = 0.47, *P* = 0.025). Findings were also consistent when using the original self-reported versus the cotinine-validated measure for past 24-hour use (see Supplementary Tables S6 and S7).

## Discussion

The findings of the current study indicate different patterns for biomarkers of exposure among youth who vape and smoke. Our first hypothesis was supported; those who smoked, whether exclusively or alongside vaping, had very similar levels of exposure to TSNA and VOCs and were exposed to higher levels of TSNA and most VOCs compared with those who exclusively vaped or did not vape or smoke. Our second hypothesis was partially supported. As hypothesized, TSNA biomarker levels among those who vaped exclusively were indistinguishable from levels among those who did not vape or smoke. There was also evidence that biomarkers for two VOCs (toluene, and acrylamide only in sensitivity analysis) were higher among youth who exclusively vaped compared with youth who neither vaped nor smoked. However, there was no evidence of difference in exposure to the VOCs acrolein and acrylonitrile between exclusive vaping and no use. Our third hypothesis was also supported; youth in the United States who smoked (dual or exclusive) had higher levels of NNAL than those in Canada or England.

Compared with those who smoked or dual used, youth who exclusively vaped had lower levels of exposure to most VOCs (acrolein, acrylamide, and acrylonitrile), except for toluene, which was higher among youth who vaped compared with dual use. Youth who vaped also had greater exposure to toluene compared with no use. High levels of toluene have been detected in vaping liquids and aerosols (7, 34), and observational studies of adults have also found higher levels of toluene exposure from vaping compared with no use and similar levels between vaping and smoking (35). Furthermore, the biomarker used for toluene exposure, BzMA, may also indicate exposure to other chemicals containing a benzyl group, such as benzyl alcohol (36) or other flavoring chemicals used in e-cigarettes (37, 38). A recent systematic review compared adults that vape with those not vaping or smoking and concluded higher biomarker levels for acrylamide and acrylonitrile, with mixed results for biomarkers of other VOCs (2, 16). Our finding that exposure to acrylamide was higher for exclusive vaping versus no use when vaping was defined as past 24-hour use, but not past-week use, may reflect the relatively short half-life (on the order of hours; ref. 39) of most urinary VOC metabolites, including acrylamide, for which some studies indicate a half-life as short as 2 hours (31, 40). For biomarkers of benzene (PhMA) and xylene (24MPhMA), virtually all of the samples in each of the vaping/smoking status groups had levels below the LOQ; hence, differences between groups were not tested. Other studies have also found a low frequency of detection for xylene metabolites (41, 42).

With regard to TSNAs, exclusive vaping was associated with lower levels of NNK exposure compared with dual use and exclusive smoking, consistent with previous studies among adults (15). In addition, the lack of a difference in NNK between exclusive vaping and no use in this study is consistent with previous work that found TSNA levels in vaping liquids at far lower levels than typically found in tobacco products (7).

Overall, the findings are generally consistent with existing reviews indicating lower exposure to most toxicants from vaping compared with smoking and similar or higher exposure to toxicants from vaping compared with neither smoking nor vaping (2, 9). Detailed comparisons with previous studies are limited by methodologic differences with the small number of studies conducted to date among youth. For example, compared with Chaffee and colleagues (18), the current study yielded higher NNAL levels for exclusive smoking, vaping, and dual use; however, the Chaffee and colleagues (18) study was conducted with a younger sample in which daily smoking was almost absent (<1% of the sample) and no participants vaped daily. Rubenstein and colleagues (19) found stronger associations between past 24-hour vaping and elevated levels of five VOCs compared with no-use controls, including exposure to acrylonitrile, acrolein, and acrylamide (tested in our study). In terms of differences in exposure between adults and youth, a recent review which identified only four studies among youth found broadly similar results overall for VOC and TSNA exposure (2). However, there remains insufficient evidence from youth who vape to provide robust comparisons with adults who vape, most of whom formerly smoked.

Country-level differences were observed for NNK among youth who smoked (dual or exclusive); those in the United States had higher levels of NNK exposure than those in Canada or England. These findings are consistent with evidence of higher NNK exposure among adults who smoke in the United States compared with Canada (21, 43). Higher levels of exposure in the United States are most likely due to differences in tobacco blend, with higher TSNA levels in US blends and lower levels in the Virginia flue-cured tobacco that dominates the Canadian and UK markets (44). TSNAs are predominantly formed during the curing and processing of tobacco and can be reduced by modifying manufacturing practices (3, 6, 45). The current findings indicate that higher levels of TSNA exposure among smokers in the United States are observable at the early stages of smoking among youth, which would translate to very large differences in exposure to a potent class of lung carcinogens over the lifetime of a long-term smoker. The industry’s ongoing failure to adopt manufacturing practices to reduce the excess risk of their products undermines their publicly stated commitment to reducing the harm of tobacco products (46).

### Limitations

The current study is subject to general limitations associated with BoEs. All biomarkers are subject to potential confounders, and some are not specific to only tobacco, such as the presence of some VOCs in household products like paints and cosmetics (33). As noted above, we were unable to compare exposure to benzene and xylene by vaping/smoking status, thus reducing the scope of VOCs about which the current study could draw conclusions. In addition, the current study used self-collection of urine samples. Although the method has previously been validated (26), protocol deviations may have occurred that could affect the accuracy of estimates, although any impact on the findings is unclear. Other measures are also subject to the limitations of self-report, including the use of cannabis and other tobacco products. Categorization of smoking and vaping status based on the past week is an appropriate time frame for estimating recent exposure but does not fully account for an individual’s smoking and vaping history, which can be highly variable among young people. Indeed, nearly one third of study participants were classified into different past-week vaping and smoking status groups between eligibility screening and the time of sample collection. Prospective cohort studies capable of capturing changes in use over time and estimating “accumulated” or “aggregate” exposure would be particularly beneficial. Approximately two thirds of the data collection occurred after the onset of the COVID-19 pandemic. The extent to which pandemic restrictions on retail stores, schools, and social gatherings may have affected patterns of consumption and biomarker levels remains unclear. Finally, the current study examined biomarkers for only a subset of constituents in tobacco smoke and vaping aerosol. The pattern of results between vaping, smoking, dual use and nonuse may differ for constituents not assessed in the study, such as some heavy metals, for which exposure may be higher among people who vape compared with nonusers and people who smoke (17). The sensitivity analysis that used cotinine values to verify smoking/vaping categories also had some limitations, as this method would exclude both people vaping low or no nicotine products from the vaping group (despite their exposure to vaping aerosols) as well as people using other nicotine-containing products (e.g., nicotine pouches) from the “no use” comparison group; using cotinine cannot differentiate nicotine sources (e.g., vaping vs. tobacco smoking), and verification using a combination of biomarkers (e.g., cotinine and NNAL) to differentiate the use of tobacco-containing products from other nicotine products may be useful in future studies (31).

### Conclusions

Exposure to toxicants measured in the study was comparable between youth that exclusively vaped and youth who neither vaped nor smoked for a TSNA and two VOCs (acrolein and acrylonitrile) and higher for one VOC (toluene; additionally for the VOC acrylamide for past 24-hour use only). Exposure to a TSNA and three VOCs (acrolein, acrylamide, and acrylonitrile) was lower among youth who exclusively vaped e-cigarettes compared with those who smoked (exclusively or in addition to vaping), with higher exposure for exclusive vaping compared with dual use for one VOC (toluene). Collectively, the findings are consistent with the current consensus that vaping is associated with reduced exposure to biomarkers of toxic constituents compared with smoking but higher levels of exposure for some toxicants compared with nonuse. Among youth who smoked, higher exposure to a TSNA—a potent lung carcinogen—among youth in the United States (vs. Canada or England) is also notable for the industry’s failure to implement manufacturing practices that are known to reduce TSNA levels in tobacco.

## Supplementary Material

Supplementary Figure 1 Flowchart for participation and eligibility in biomarker study

Table S1 Participant characteristics at time of sample collection, overall and by country

Table S2 Biomarkers of exposure within past-24-hour smoking/vaping status groups, n(%) samples with concentration above LLOQ and geometric means (SD) concentration, normalized for mg creatinine

Table S3 Comparisons between past-24-hour smoking/vaping status groups for biomarkers of exposure, ng/ml

Table S4 Biomarkers of exposure within past-week vaping and tobacco smoking status groups (cotinine-validated), n(%) samples with concentration above LLOQ and geometric mean (SD) concentrations, normalized for mg creatinine

Table S5 Comparisons between past-week vaping and tobacco smoking status groups (cotinine-validated) for biomarkers of exposure, ng/mg

Table S6 Biomarkers of exposure within past-24-hour smoking/vaping status groups (cotinine-validated), n(%) samples with concentration above LOQ and geometric means (SD) concentration, normalized for mg creatinine

Table S7 Comparisons between past-24-hour smoking/vaping status groups (cotinine-validated) for biomarkers of exposure, ng/ml

Supplementary Materials and Methods Participant Questionnaires
