# Supplementary material for: Biomarkers of Toxicant Exposure among Youth in Canada, England, and the United States Who Vape and/or Smoke Tobacco or Do Neither
Source: Cancer Epidemiol Biomarkers Prev. 2025 Feb 24;34(5):815–24. doi: 10.1158/1055-9965.EPI-24-1338 (PMC12046313; doi:10.1158/1055-9965.EPI-24-1338)
Supplement: Supplementary Figure 1 — Flowchart for participation and eligibility in biomarker study [file epi-24-1338_supplementary_figure_1_suppsf1.pdf]

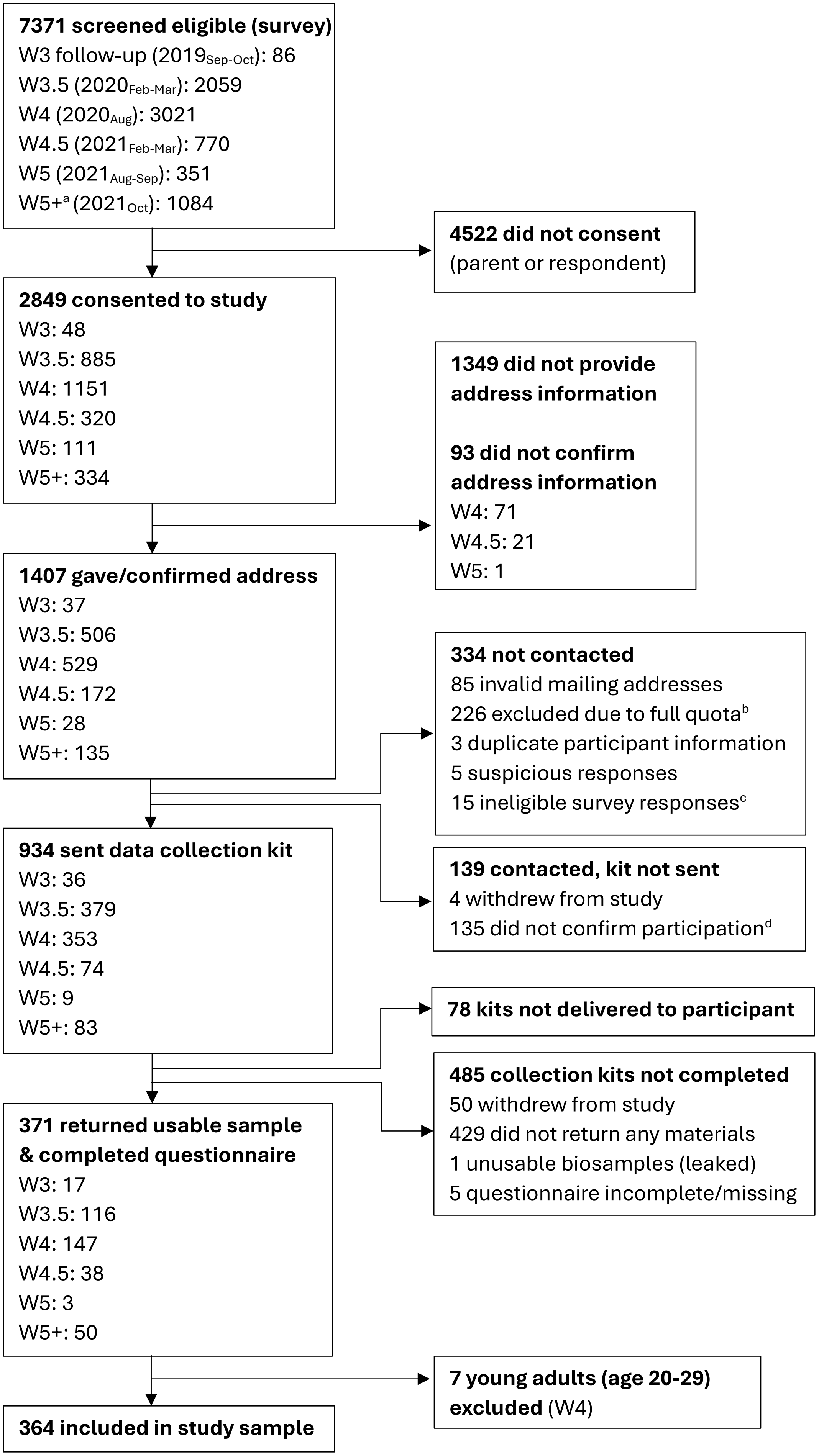

Figure S1: Flowchart for participation and eligibility in biomarker study
Eligibility and participation in the study are shown by wave of recruitment.
[illegible]

Abbreviations: W=Wave

Notes:

[a]W5+ was supplementary data collection in Canada only, conducted by Leger

[b]In W3.5 and W4, the ‘non-user’ groups were capped at 40 per country; no ‘non-users’ were recruited in W4.5, W5, W5+; no participants were contacted in England in W5

[c]In W5+, respondents who agreed to participant were asked to complete the W5 survey: 12 gave responses that indicated ineligible user status and 3 failed the data quality check question

[d]In W4.5 and W5, respondents were contacted and asked to confirm their participation before kits were sent
