# Supplementary material for: Biomarkers of Toxicant Exposure among Youth in Canada, England, and the United States Who Vape and/or Smoke Tobacco or Do Neither
Source: Cancer Epidemiol Biomarkers Prev. 2025 Feb 24;34(5):815–24. doi: 10.1158/1055-9965.EPI-24-1338 (PMC12046313; doi:10.1158/1055-9965.EPI-24-1338)
Supplement: Table S1 — Participant characteristics at time of sample collection, overall and by country [file epi-24-1338_table_s1_suppst1.pdf]

Table S1. Participant characteristics at time of sample collection, overall and by country (n=364)

	Canada n=129	England n=131	US n=104	Total N=364
	n (%)	n (%)	n (%)	n (%)
**Age** (mean, SD)	17.61 (1.09)	17.62 (1.09)	17.38 (1.05)	17.55 (1.08)
**Sex**				
Male	59 (45.7%)	56 (42.7%)	46 (44.2%)	161 (44.2%)
Female	70 (54.3%)	75 (57.3%)	58 (55.8%)	203 (55.8%)
**Past-week smoking/vaping**				
No use	52 (40.3%)	57 (43.5%)	37 (35.6%)	146 (40.1%)
Vaping (exclusive)	35 (27.1%)	14 (10.7%)	24 (23.1%)	73 (20.1%)
Tobacco smoking[a] (exclusive)	16 (12.4%)	33 (25.2%)	19 (18.3%)	68[c] (18.7%)
Vaping and smoking (dual use)[a]	26 (20.2%)	27 (20.6%)	24 (23.1%)	77[d] (21.2%)
**Past-24h smoking/vaping**				
No use	64 (49.6%)	73 (55.7%)	48 (46.2%)	185 (50.8%)
Vaping (exclusive)	34 (26.4%)	14 (10.7%)	22 (21.2%)	70 (19.2%)
Tobacco smoking[a] (exclusive)	15 (11.6%)	28 (21.4%)	15 (14.4%)	58 (15.9%)
Vaping and smoking (dual use)[a]	16 (12.4%)	16 (12.2%)	19 (18.3%)	51 (14.0%)
**Past-week cannabis use**				
No use	78 (60.5%)	112 (85.5%)	71 (68.3%)	261 (71.7%)
Cannabis vaping (exclusive)	1 (0.8%)	3 (2.3%)	4 (3.8%)	8 (2.2%)
Cannabis smoking (exclusive)	36 (27.9%)	13 (9.9%)	20 (19.2%)	69 (19.0%)
Cannabis vaping and smoking	13 (10.1%)	3 (2.3%)	9 (8.7%)	25 (6.9%)
Missing	1 (0.8%)	--	--	1 (0.3%)
**Past-week smokeless tobacco use**				
No	126 (97.7%)	128 (97.7%)	103 (99.0%)	357 (98.1%)
Yes	3 (2.3%)	3 (2.3%)	1 (1.0%)	7 (1.9%)
**Past-week NRT use**				
No	126 (97.7%)	122 (93.1%)	103 (99.0%)	351 (96.4%)
Yes	3 (2.3%)	8 (6.1%)	1 (1.0%)	12 (3.3%)
Missing	--	1 (0.8%)	--	1 (0.3%)
**Past-week SHS exposure[b]**				
No	40 (31.0%)	45 (34.4%)	35 (33.7%)	120 (33.0%)
Yes	46 (35.7%)	34 (26.0%)	35 (33.7%)	115 (31.6%)
Missing	43 (33.3%)	52 (39.7%)	34 (32.7%)	129 (35.4%)

[a]Includes cigarettes and other smoked tobacco (cigar, cigarillo, bidi, shisha, etc.).

[b]Question about SHS added at Wave 4 (2020_{Aug}).

[c]Of the 68 who exclusively smoked, n=53 smoked cigarettes but not other tobacco, n=9 smoked both cigarettes and other tobacco, n=4 smoked other tobacco but not cigarettes, and n=2 smoked cigarettes but other tobacco was unknown.

[d]Of the 77 who exclusively smoked, n=57 smoked cigarettes but not other tobacco, n=16 smoked both cigarettes and other tobacco, and n=4 smoked other tobacco but not cigarettes.

Abbreviations: NRT, nicotine replacement therapy; SHS, second-hand smoke
