# Supplementary material for: Biomarkers of Toxicant Exposure among Youth in Canada, England, and the United States Who Vape and/or Smoke Tobacco or Do Neither
Source: Cancer Epidemiol Biomarkers Prev. 2025 Feb 24;34(5):815–24. doi: 10.1158/1055-9965.EPI-24-1338 (PMC12046313; doi:10.1158/1055-9965.EPI-24-1338)
Supplement: Table S2 — Biomarkers of exposure within past-24-hour smoking/vaping status groups, n(%) samples with concentration above LLOQ and geometric means (SD) concentration, normalized for mg creatinine [file epi-24-1338_table_s2_suppst2.pdf]

Table S2: Biomarkers of exposure within past-24-hour smoking/vaping status groups, n(%) samples with concentration above LLOQ and geometric means (SD) concentration, normalized for mg creatinine

	TSNA	VOC Biomarkers					
	**NNK (NNAL)**	**Acrolein (3HPMA)**	**Acrylamide (2CaHEMA)**	**Acrylonitrile (2CyEMA)**	**Benzene (PhMA)**	**Toluene (BzMA)**	**Xylene (24MPhMA)**
**LLOQ**	3.0pg/mL	30.0ng/mL	10.0ng/mL	5.0ng/mL	2.0ng/mL	2.0ng/mL	2.0ng/mL
**PRESENCE** n present/ total (%)							
**No use**	35/185 (18.9%)	182/185 (98.4%)	159/185 (85.9%)	84/185 (45.4%)	0/185 (0%)	160/185 (86.5%)	0/185 (0%)
**Past-24h vaping (exclusive)**	18/70 (25.7%)	70/70 (100%)	67/70 (95.7%)	49/70 (70.0%)	2/70 (2.9%)	64/70 (91.4%)	0/70 (0%)
**Past-24h smoking (exclusive)**	52/58 (89.7%)	58/58 (100%)	56/58 (96.6%)	53/58 (91.4%)	2/58 (3.4%)	56/58 (96.6%)	0/58 (0%)
**Dual use**	41/51 (80.4%)	51/51 (100%)	49/51 (96.0%)	45/51 (88.2%)	2/51 (3.9%)	41/51 (80.4%)	0/51 (0%)
**CONCENTRATION**[a] geometric mean (SD)	pg/mg	ng/mg	ng/mg	ng/mg	ng/mg	ng/mg	ng/mg
**No use**	2.28 (6.64)	326.1 (515. 7)	19.6 (14.2)	3.9 (30.9)	n/a[b]	3.97 (4.79)	n/a[b]
**Past-24h vaping (exclusive)**	2.15 (8.50)	345.1 (294.4)	24.7 (22.1)	5.3 (39.5)	n/a[b]	4.39 (9.41)	n/a[b]
**Past-24h smoking (exclusive)**	34.84 (104.70)	832.3 (1164.8)	41.3 (30.3)	49.2 (90.4)	n/a[b]	5.00 (4.21)	n/a[b]
**Dual use**	20.92 (72.04)	740.5 (1458.4)	39.6 (169.6)	33.7 (91.2)	n/a[b]	3.83 (4.00)	n/a[b]

[a]Estimates of concentration exclude outliers (n=4 for 3HPMA; n=7 for 2CaHEMA; n=6 for 2CyEMA; n=8 for BzMA; n=6 for NNAL), participants with creatinine values outside of the reference range (n=3), and samples where the sample matrix affected accurate detection of results (n=1 for 3HPMA; n=15 for 2CaHEMA; n=1 for 2CyEMA; n=1 for BzMA; n=3 for NNAL). For NNAL, n=1 value <LLOQ cut-off but quantified was included.

[b]>95% of samples had levels below the level of quantitation
