# Supplementary material for: Biomarkers of Toxicant Exposure among Youth in Canada, England, and the United States Who Vape and/or Smoke Tobacco or Do Neither
Source: Cancer Epidemiol Biomarkers Prev. 2025 Feb 24;34(5):815–24. doi: 10.1158/1055-9965.EPI-24-1338 (PMC12046313; doi:10.1158/1055-9965.EPI-24-1338)
Supplement: Table S3 — Comparisons between past-24-hour smoking/vaping status groups for biomarkers of exposure, ng/ml [file epi-24-1338_table_s3_suppst3.pdf]

Table S3: Comparisons between past-24-hour smoking/vaping status groups[a] for biomarkers of exposure, ng/ml

	Smoking/vaping status	Vaped vs No use	Smoked vs No use	Dual use vs No use	Vaped vs Smoked	Vaped vs Dual use	Smoked vs Dual use
	**Model effect**	**B [95%CI] (p value) for comparison**					
**NNAL** (n=351)	**Wald X^2=306.0 (p<0.001)**	0.08 [-0.23,0.38] (p=0.63)	**2.53 [2.20,2.85] (p<0.001)**	**2.00 [1.64,2.36] (p<0.001)**	**-2.45 [-2.83,-2.07] (p<0.001)**	**-1.93 [-2.32,-1.53] (p<0.001)**	**0.53 [0.12,0.93] (p=0.011)**
**3HPMA** (n=355)	**Wald X^2=65.3 (p<0.001)**	0.08 [-0.17,0.32] (p=0.54)	**0.89 [0.63,1.14] (p<0.001)**	**0.81 [0.52,1.10] (p<0.001)**	**-0.81 [-1.11,-0.51] (p<0.001)**	**-0.74 [-1.05,-0.42] (p<0.001)**	0.08 [-0.25,0.40] (p=0.65)
**2CaHEMA** (n=338)	**Wald X^2=50.3 (p<0.001)**	**0.20 [0.15,0.38] (p=0.034)**	**0.62 [0.43,0.80] (p<0.001)**	**0.53 [0.31,0.74] (p<0.001)**	**-42 [-0.64,-0.19] (p<0.001)**	**-0.33 [-0.56,-0.09] (p=0.006)**	0.09 [-0.15,0.33] (p=0.47)
**2CyEMA** (n=353)	**Wald X^2=204.9 (p<0.001)**	0.29 [-0.32,0.61] (p=0.078)	**2.20 [1.87,2.53] (p<0.001)**	**1.69 [1.31,2.07] (p<0.001)**	**-1.91 [-2.30,-1.52] (p<0.001)**	**-1.40 [-1.81,-1.00] (p<0.001)**	**0.51 [0.09,0.93] (p=0.018)**
**BzMA** (n=351)	Wald X^2=7.5 (p=0.059)	**0.23 [0.02,0.44] (p=0.036)**	0.15 [-0.72,0.36] (p=0.19)	-0.08 [-0.33,0.17] (p=0.54)	0.08 [-0.18,0.34] (p=0.54)	**0.30 [0.03,0.58] (p=0.027)**	0.22 [-0.05,0.50] (p=0.11)

Bolded values indicate statistical significance at the p<0.05 level

[a]From separate linear regression models for each biomarker (using log transformed values) adjusted for creatinine, age, sex, country, and cannabis use in the past 7 days (no use, exclusive vaping, exclusive smoking, both vaping and smoking)
