# Supplementary material for: Biomarkers of Toxicant Exposure among Youth in Canada, England, and the United States Who Vape and/or Smoke Tobacco or Do Neither
Source: Cancer Epidemiol Biomarkers Prev. 2025 Feb 24;34(5):815–24. doi: 10.1158/1055-9965.EPI-24-1338 (PMC12046313; doi:10.1158/1055-9965.EPI-24-1338)
Supplement: Table S4 — Biomarkers of exposure within past-week vaping and tobacco smoking status groups (cotinine-validated), n(%) samples with concentration above LLOQ and geometric mean (SD) concentrations, normalized for mg creatinine [file epi-24-1338_table_s4_suppst4.pdf]

Table S4. Biomarkers of exposure within past-week vaping and tobacco smoking status groups (cotinine-validated[a]), n(%) samples with concentration above LLOQ and geometric mean (SD) concentrations, normalized for mg creatinine

	TSNA	VOC Biomarkers					
	**NNK (NNAL)**	**Acrolein (3HPMA)**	**Acrylamide (2CaHEMA)**	**Acrylonitrile (2CyEMA)**	**Benzene (PhMA)**	**Toluene (BzMA)**	**Xylene (24MPhMA)**
**LLOQ**	3.0pg/mL	30.0ng/mL	10.0ng/mL	5.0ng/mL	2.0ng/mL	2.0ng/mL	2.0ng/mL
**PRESENCE** n present/ total (%)							
**No use**	24/143 (16.8%)	141/143 (98.6%)	124/143 (86.7%)	60/143 (42.0%)	0/143 (0%)	129/143 (90.2%)	0/143 (0%)
**Past-week vaping**	12/52 (23.1%)	52/52 (100%)	51/52 (98.1%)	37/52 (71.2%)	1/52 (1.9%)	48/52 (92.3%)	0/52 (0%)
**Past-week smoking**	46/49 (93.9%)	49/49 (100%)	48/49 (98.0%)	47/49 (95.9%)	2/49 (4.1%)	45/49 (91.8%)	0/49 (0%)
**Dual use (past-week vaping and smoking)**	52/61 (85.2%)	61/61 (100%)	57/61 (93.4%)	57/61 (93.4%)	3/61 (4.9%)	49/61 (80.3%)	0/61 (0%)
**CONCENTRATION[b]** geometric mean (SD)	pg/mg	ng/mg	ng/mg	ng/mg	ng/mg	ng/mg	ng/mg
**No use**	2.1 (2.6)	324.0 (302.6)	19.0 (11.2)	3.5 (4.9)	n/a[c]	3.96 (4.09)	n/a[c]
**Past-week vaping**	1.9 (5.2)	354.0 (309.5)	24.6 (16.6)	5.6 (42.1)	n/a[c]	4.38 (9.22)	n/a[c]
**Past-week smoking**	38.0 (98.0)	827.4 (1382.8)	42.0 (29.5)	54.6 (102.5)	n/a[c]	4.71 (4.02)	n/a[c]
**Dual use (past-week vaping and smoking)**	23.7 (83.4)	746.3 (1399.8)	37.1 (156.4)	38.6 (86.8)	n/a[c]	3.94 (4.27)	n/a[c]

Abbreviations: TSNA, tobacco-specific nitrosamine; VOC, volatile organic compound; LLOQ, lowest limit of quantitation

[a]Validation of self-reported past-week vaping and tobacco smoking status consisted of excluding those in the 'no use' category who had cotinine values above 50ng/mg creatinine (n=3), and those in the vaping, smoking or dual use groups whose cotinine values were below the LLOQ of 5ng/mL (n=56: n=21 vaping, n=19 smoking, n=15 dual use).

[b]Estimates of concentration exclude outliers (n=3 for 3HPMA; n=5 for 2CaHEMA; n=4 for 2CyEMA; n=7 for BzMA; n=5 for NNAL), participants with creatinine values outside of the reference range (n=3), and samples where the sample matrix affected accurate detection of results (n=1 for 3HPMA; n=11 for 2CaHEMA; n=1 for 2CyEMA; n=1 for BzMA; n=2 for NNAL). For NNAL, n=1 value <LLOQ cut-off but quantified was included.

[c]>95% of samples had levels below the limit of quantitation
