# Supplementary material for: Biomarkers of Toxicant Exposure among Youth in Canada, England, and the United States Who Vape and/or Smoke Tobacco or Do Neither
Source: Cancer Epidemiol Biomarkers Prev. 2025 Feb 24;34(5):815–24. doi: 10.1158/1055-9965.EPI-24-1338 (PMC12046313; doi:10.1158/1055-9965.EPI-24-1338)
Supplement: Table S5 — Comparisons between past-week vaping and tobacco smoking status groups (cotinine-validated) for biomarkers of exposure, ng/mg [file epi-24-1338_table_s5_suppst5.pdf]

Table S5: Comparisons between past-week vaping and tobacco smoking status groups[a] (cotinine-validated) for biomarkers of exposure, ng/mg

	Vaping/Smoking status	Vaped vs No use	Smoked vs No use	Dual use vs No use	Vaped vs Smoked	Vaped vs Dual use	Smoked vs Dual use
	**Model effect**	**B [95%CI] (p value) for comparison**					
**NNAL[b]** (n=294)	**Wald X^2=360.8 (p<0.001)**	0.02 [-0.33,0.37] (p=0.90)	**2.70 [2.36,3.04] (p<0.001)**	**1.88 [1.88,2.56] (p<0.001)**	**-2.68 [-3.09,-2.27] (p<0.001)**	**-2.20 [-2.58,-1.82] (p<0.001)**	**0.48 [0.10,0.86] (p=0.014)***
**3HPMA** (n=297)	**Wald X^2=57.5 (p<0.001)**	0.12 [-0.18,0.43] (p=0.42)	**0.91 [0.62,1.20] (p<0.001)**	**0.83 [0.54,1.13] (p<0.001)**	**-0.78 [-1.13,0.43] (p<0.001)**	**-0.71 [-1.03,-0.39] (p<0.001)**	0.07 [-0.26,0.40] (p=0.67)
**2CaHEMA** (n=285)	**Wald X^2=47.2 (p<0.001)**	0.19 [-0.04,0.41] (p=0.11)	**0.66 [0.46,0.87] (p<0.001)**	**0.51 [0.29-0.72] (p<0.001)**	**-0.48 [-0.73,-0.22] (p<0.001)**	**-0.32 [-0.56,-0.08] (p=0.010)**	0.16 [-0.08,0.39] (p=0.20)
**2CyEMA** (n=296)	**Wald X^2=228.7 (p<0.001)**	**0.40 [0.03,0.78] (p=0.034)***	**2.40 [2.04-2.75] (p<0.001)**	**1.93 [1.57,2.29] (p<0.001)**	**-1.99 [-2.43,-1.56] (p<0.001)**	**-1.53 [-1.93,-1.12] (p<0.001)**	**0.47 [0.06,0.88] (p=0.025)***
**BzMA** (n=293)	Wald X^2=4.3 (p=0.23)	0.23 [-0.03,0.48] (p=0.077)*	0.03 [-0.17,0.32] (p=0.56)	-0.08 [-0.27,0.22] (p=0.83)	0.16 [-0.14,0.45] (p=0.30)	0.26 [-0.02,0.53] (p=0.066)*	0.10 [-0.18,0.37] (p=0.48)

Bolded values indicate statistical significance at the p<0.05 level; * indicates a difference in significance level compared to the model based on self-reported measure

[a]From separate linear regression models for each biomarker (using log transformed values) adjusted for creatinine, age, sex, country, and cannabis use in the past 7 days (no use, exclusive vaping, exclusive smoking, both vaping and smoking)

[b]pg/mg creatinine
