# Supplementary material for: Biomarkers of Toxicant Exposure among Youth in Canada, England, and the United States Who Vape and/or Smoke Tobacco or Do Neither
Source: Cancer Epidemiol Biomarkers Prev. 2025 Feb 24;34(5):815–24. doi: 10.1158/1055-9965.EPI-24-1338 (PMC12046313; doi:10.1158/1055-9965.EPI-24-1338)
Supplement: Table S6 — Biomarkers of exposure within past-24-hour smoking/vaping status groups (cotinine-validated), n(%) samples with concentration above LOQ and geometric means (SD) concentration, normalized for mg creatinine [file epi-24-1338_table_s6_suppst6.pdf]

Table S6: Biomarkers of exposure within past-24-hour smoking/vaping status groups (cotinine-validated[a]), n(%) samples with concentration above LOQ and geometric means (SD) concentration, normalized for mg creatinine

	TSNA	VOC Biomarkers					
	**NNK (NNAL)**	**Acrolein (3HPMA)**	**Acrylamide (2CaHEMA)**	**Acrylonitrile (2CyEMA)**	**Benzene (PhMA)**	**Toluene (BzMA)**	**Xylene (24MPhMA)**
**LLOQ**	3.0pg/mL	30.0ng/mL	10.0ng/mL	5.0ng/mL	2.0ng/mL	2.0ng/mL	2.0ng/mL
**PRESENCE** n present/ total (%)							
**No use**	35/176 (18.9%)	174/177 (98.3%)	151/177 (85.3%)	77/177 (43.5%)	0/176 (0%)	154/177 (87.0%)	0/177 (0%)
**Past-24h vaping (exclusive)**	18/58 (25.7%)	58/58 (100%)	55/58 (94.8%)	41/58 (70.7%)	2/58 (3.4%)	52/58 (89.7%)	0/58 (0%)
**Past-24h smoking (exclusive)**	52/48 (89.7%)	48/48 (100%)	47/48 (97.9%)	48/48 (100%)	2/48 (4.2%)	47/48 (97.9%)	0/48 (0%)
**Dual use**	41/43 (80.4%)	43/43 (100%)	43/43 (100%)	43/43 (100%)	2/43 (4.7%)	35/43 (81.4%)	0/43 (0%)
**CONCENTRATION[b]** geometric mean (SD)	pg/mg	ng/mg	ng/mg	ng/mg	ng/mg	ng/mg	ng/mg
**No use**	2.16 (2.78)	321.4 (302.5)	19.2 (13.5)	3.7 (5.9)	n/a[c]	3.95 (4.80)	n/a[c]
**Past-24h vaping (exclusive)**	2.25 (9.29)	350.7 (312.8)	25.2 (23.8)	6.0 (43.1)	n/a[c]	4.26 (8.79)	n/a[c]
**Past-24h smoking (exclusive)**	46.32 (109.46)	945.1 (1215.7)	42.9 (27.2)	70.2 (91.0)	n/a[c]	4.87 (4.38)	n/a[c]
**Dual use**	33.14 (74.23)	907.1 (1520.9)	41.9 (182.7)	53.0 (92.7)	n/a[c]	3.92 (4.11)	n/a[c]

[a]Validation of self-reported past-24-hour vaping and tobacco smoking status consisted of excluding those in the 'no use' category who had cotinine values above 50ng/mg creatinine (n=8), and those in the vaping, smoking or dual use groups whose cotinine values were below the LLOQ of 5ng/mL (n=30: n=12 vaping, n=10 smoking, n=8 dual use).

[b]Estimates of concentration exclude outliers (n=3 for 3HPMA; n=6 for 2CaHEMA; n=4 for 2CyEMA; n=7 for BzMA; n=5 for NNAL) and samples where the sample matrix affected accurate detection of results (n=1 for 3HPMA; n=11 for 2CaHEMA; n=1 for 2CyEMA; n=1 for BzMA; n=2 for NNAL). For NNAL, n=1 value <LLOQ cut-off but quantified was included.

[c]>95% of samples had levels below the limit of quantitation
