# Supplementary material for: Biomarkers of Toxicant Exposure among Youth in Canada, England, and the United States Who Vape and/or Smoke Tobacco or Do Neither
Source: Cancer Epidemiol Biomarkers Prev. 2025 Feb 24;34(5):815–24. doi: 10.1158/1055-9965.EPI-24-1338 (PMC12046313; doi:10.1158/1055-9965.EPI-24-1338)
Supplement: Table S7 — Comparisons between past-24-hour smoking/vaping status groups (cotinine-validated) for biomarkers of exposure, ng/ml [file epi-24-1338_table_s7_suppst7.pdf]

Table S7: Comparisons between past-24-hour smoking/vaping status groups[a] (cotinine-validated) for biomarkers of exposure, ng/ml

	Smoking/vaping status	Vaped vs No use	Smoked vs No use	Dual use vs No use	Vaped vs Smoked	Vaped vs Dual use	Smoked vs Dual use
	**Model effect**	**B [95%CI] (p value) for comparison**					
**NNAL[b]** (n=315)	**Wald X^2=523.2 (p<0.001)**	0.26 [-0.03-0.56] (p=0.077)	**3.00 [2.70,3.30] (p<0.001)**	**2.62 [2.29-2.96] (p<0.001)**	**-2.73 [-3.09,-2.38] (p<0.001)**	**-2.36 [-2.72,-2.00] (p<0.001)**	0.37 [-0.01,0.75] (p=0.053)*
**3HPMA** (n=318)	**Wald X^2=97.0 (p<0.001)**	0.17 [-0.10,0.43] (p=0.21)	**1.11 [0.84,1.38] (p<0.001)**	**1.09 [0.79,1.39] (p<0.001)**	**-0.94 [-1.27,-0.62] (p<0.001)**	**-0.92 [-1.25,-0.59] (p<0.001)**	0.02 [-0.32,0.37] (p=0.89)
**2CaHEMA** (n=305)	**Wald X^2=59.2 (p<0.001)**	**0.22 [0.01-0.42] (p=0.037)**	**0.70 [0.50-0.90] (p<0.001)**	**0.61 [0.38-0.84] (p<0.001)**	**-0.49 [-0.73,-0.24] (p<0.001)**	**-0.39 [-0.64,-0.14] (p=0.003)**	0.10 [-0.16,0.35] (p=0.47)
**2CyEMA** (n=317)	**Wald X^2=372.5 (p<0.001)**	**0.52 [0.22,0.83] (p<0.001)***	**2.72 [2.40,3.03] (p<0.001)**	**2.27 [1.92,2.62] (p<0.001)**	**-2.20 [-2.57,-1.82] (p<0.001)**	**-1.75 [-2.13,-1.37] (p<0.001)**	**0.44 [0.05,0.84] (p=0.028)**
**BzMA** (n=314)	Wald X^2=5.6 (p=0.13)	0.21 [-0.03,0.44] (p=0.085)*	0.18 [-0.05,0.42] (p=0.13)	-0.04 [-0.31,0.23] (p=0.76)	0.02 [-0.26,0.31] (p=0.87)	0.25 [-0.04,0.54] (p=0.095)*	0.23 [-0.08,0.53] (p=0.14)

Bolded values indicate statistical significance at the p<0.05 level; * indicates a difference in significance level compared to the model based on self-reported measure

[a]From separate linear regression models for each biomarker (using log transformed values) adjusted for creatinine, age, sex, country, and cannabis use in the past 7 days (no use, exclusive vaping, exclusive smoking, both vaping and smoking)

[b]pg/mg creatinine
