# Supplementary material for: Biomarkers of Toxicant Exposure among Youth in Canada, England, and the United States Who Vape and/or Smoke Tobacco or Do Neither
Source: Cancer Epidemiol Biomarkers Prev. 2025 Feb 24;34(5):815–24. doi: 10.1158/1055-9965.EPI-24-1338 (PMC12046313; doi:10.1158/1055-9965.EPI-24-1338)
Supplement: Supplementary Materials and Methods — Participant Questionnaires [file epi-24-1338_supplementary_materials_and_methods_suppsm1.pdf]

# SUPPLEMENT: PARTICIPANT QUESTIONNAIRES

**IMPORTANT: PLEASE FILL THIS OUT IMMEDIATELY AFTER COLLECTING YOUR URINE SAMPLE.**

For the urine sample quality control purposes, please answer these essential questions:

DATE OF COLLECTION: _______________ TIME OF COLLECTION _______________ am / pm

**When was the LAST time you did the following…**

*Please circle one response per product (row).*

| | | | | | | |
|---|---|---|---|---|---|---|
| **Used an e-cigarette/vaped** | Less than 1 hour ago | 1-6 hours ago | 7-12 hours ago | 12-24 hours ago | 1-7 days ago | Not at all in last 7 days |
| **Smoked a regular cigarette** | Less than 1 hour ago | 1-6 hours ago | 7-12 hours ago | 12-24 hours ago | 1-7 days ago | Not at all in last 7 days |
| **Smoked any other tobacco** (cigar, cigarillo, bidi, shisha, etc.) | Less than 1 hour ago | 1-6 hours ago | 7-12 hours ago | 12-24 hours ago | 1-7 days ago | Not at all in last 7 days |
| **Smoked cannabis/ marijuana** | Less than 1 hour ago | 1-6 hours ago | 7-12 hours ago | 12-24 hours ago | 1-7 days ago | Not at all in last 7 days |
| **Vaped cannabis/ marijuana** | Less than 1 hour ago | 1-6 hours ago | 7-12 hours ago | 12-24 hours ago | 1-7 days ago | Not at all in last 7 days |
| **Used smokeless tobacco** (chew, pinch, snuff, snus) | Less than 1 hour ago | 1-6 hours ago | 7-12 hours ago | 12-24 hours ago | 1-7 days ago | Not at all in last 7 days |
| **Used nicotine replacement therapy** (patches, gum, lozenges, etc.) **or nicotine pouches** | Less than 1 hour ago | 1-6 hours ago | 7-12 hours ago | 12-24 hours ago | 1-7 days ago | Not at all in last 7 days |
| **Ate grilled meat** | Less than 1 hour ago | 1-6 hours ago | 7-12 hours ago | 12-24 hours ago | 1-7 days ago | Not at all in last 7 days |

**Please tell us more about the LAST e-cigarette you used:**

1. What brand was the device?
   (*please be as specific as possible*) ______________________________________________
2. What brand was the cartridge, pod or e-liquid?
   (*please be as specific as possible*) ______________________________________________
3. a) Did it contain nicotine? (*circle one*)

   Yes
   No
   Don’t know

   b) **If Yes**: Was it nicotine salt? (*circle one*)

   Yes
   No
   Don’t know

**PLEASE ENSURE YOU INCLUDE THIS FORM WITH YOUR URINE SAMPLES IN THE ENVELOPE PROVIDED.**

WAVE 3.5 – UPDATES

**IMPORTANT: PLEASE FILL THIS OUT IMMEDIATELY AFTER COLLECTING YOUR URINE SAMPLE.**

Note: We will NOT test for any illegal substances. Questions about cannabis are for quality control of samples.

DATE OF COLLECTION: _______________ TIME OF COLLECTION: _______________ am / pm

## When was the LAST time you did the following…

*Please circle one response per product (row). If you have never done the following, select 'Not at all in last 7 days'.*

| | | | | | | |
|---|---|---|---|---|---|---|
| **Used an e-cigarette/vaped** | Less than 1 hour ago | 1-6 hours ago | 7-12 hours ago | 12-24 hours ago | 1-7 days ago | Not at all in last 7 days |
| **Smoked a regular cigarette** | Less than 1 hour ago | 1-6 hours ago | 7-12 hours ago | 12-24 hours ago | 1-7 days ago | Not at all in last 7 days |
| **Smoked any other tobacco** (cigar, cigarillo, bidi, shisha, etc.) | Less than 1 hour ago | 1-6 hours ago | 7-12 hours ago | 12-24 hours ago | 1-7 days ago | Not at all in last 7 days |
| **Smoked cannabis/ marijuana** | Less than 1 hour ago | 1-6 hours ago | 7-12 hours ago | 12-24 hours ago | 1-7 days ago | Not at all in last 7 days |
| **Vaped cannabis/ marijuana** | Less than 1 hour ago | 1-6 hours ago | 7-12 hours ago | 12-24 hours ago | 1-7 days ago | Not at all in last 7 days |
| **Used smokeless tobacco** (chew, pinch, snuff, snus) | Less than 1 hour ago | 1-6 hours ago | 7-12 hours ago | 12-24 hours ago | 1-7 days ago | Not at all in last 7 days |
| **Used nicotine replacement therapy** (patches, gum, lozenges, etc.) **or nicotine pouches** | Less than 1 hour ago | 1-6 hours ago | 7-12 hours ago | 12-24 hours ago | 1-7 days ago | Not at all in last 7 days |
| **Ate grilled meat** (i.e, cooked over flame or charcoal, or with black grill marks) | Less than 1 hour ago | 1-6 hours ago | 7-12 hours ago | 12-24 hours ago | 1-7 days ago | Not at all in last 7 days |

## Please tell us more about the LAST e-cigarette you used *(if applicable):*

1. What brand was the device?
   (*please be as specific as possible*) ________________________________________

2. What brand was the cartridge, pod or e-liquid?
   (*please be as specific as possible*) ________________________________________

3. What flavour was the cartridge, pod or e-liquid? ________________________________________

4. a) Did it contain nicotine? (*circle one*)

   Yes →
   No
   Don't know

   b) What concentration of nicotine was it? (*answer in mg/ml OR %*)

   ________ mg/ml **OR** ______% nicotine

   Don't know

   c) Was it nicotine salt? (*circle one*)

   Yes
   No
   Don't know

**PLEASE INCLUDE THIS FORM WITH YOUR URINE SAMPLES IN THE ENVELOPE PROVIDED.**

**IMPORTANT: PLEASE FILL THIS OUT IMMEDIATELY AFTER COLLECTING YOUR URINE SAMPLE.**

Note: We will NOT test for any illegal substances. Questions about cannabis are for quality control of samples.

DATE OF COLLECTION: _______________ TIME OF COLLECTION: _______________ am / pm

## When was the LAST time you did the following…

*Please circle one response per row. If you have never done the following, select 'Not at all in last 7 days'.*

| | | | | | | |
|---|---|---|---|---|---|---|
| **Used an e-cigarette/vaped** | Less than 1 hour ago | 1-6 hours ago | 7-12 hours ago | 12-24 hours ago | 1-7 days ago | Not at all in last 7 days |
| **Smoked a regular cigarette** | Less than 1 hour ago | 1-6 hours ago | 7-12 hours ago | 12-24 hours ago | 1-7 days ago | Not at all in last 7 days |
| **Smoked any other tobacco** (cigar, cigarillo, bidi, shisha, etc.) | Less than 1 hour ago | 1-6 hours ago | 7-12 hours ago | 12-24 hours ago | 1-7 days ago | Not at all in last 7 days |
| **Smoked cannabis/marijuana** | Less than 1 hour ago | 1-6 hours ago | 7-12 hours ago | 12-24 hours ago | 1-7 days ago | Not at all in last 7 days |
| **Vaped cannabis/marijuana** | Less than 1 hour ago | 1-6 hours ago | 7-12 hours ago | 12-24 hours ago | 1-7 days ago | Not at all in last 7 days |
| **Used smokeless tobacco** (chew, pinch, snuff, snus) | Less than 1 hour ago | 1-6 hours ago | 7-12 hours ago | 12-24 hours ago | 1-7 days ago | Not at all in last 7 days |
| **Used nicotine replacement therapy** (patches, gum, lozenges, etc.) **or nicotine pouches** | Less than 1 hour ago | 1-6 hours ago | 7-12 hours ago | 12-24 hours ago | 1-7 days ago | Not at all in last 7 days |
| **Ate grilled meat** (i.e, cooked over flame or charcoal, or with black grill marks) | Less than 1 hour ago | 1-6 hours ago | 7-12 hours ago | 12-24 hours ago | 1-7 days ago | Not at all in last 7 days |
| **Were in the presence of someone smoking cigarettes or tobacco inside** (home, car, etc.) | Less than 1 hour ago | 1-6 hours ago | 7-12 hours ago | 12-24 hours ago | 1-7 days ago | Not at all in last 7 days |

## Please tell us more about the LAST e-cigarette you used *(if applicable)*:

1. **What brand was the device?**
   (*please be as specific as possible*) ______________________________________________

2. **What brand was the cartridge, pod or e-liquid?**
   (*please be as specific as possible*) ______________________________________________

3. **What flavour was the cartridge, pod or e-liquid?** ______________________________________________

4. a) **Did it contain nicotine?** (*circle one*)
   Yes →
   No
   Don't know

   b) **What concentration of nicotine was it?** (*answer in mg/ml OR %*)
   ________ mg/ml **OR** ______% nicotine
   Don't know

   c) **Was it nicotine salt?** (*circle one*)
   Yes
   No
   Don't know

**PLEASE INCLUDE THIS FORM WITH YOUR URINE SAMPLES IN THE ENVELOPE PROVIDED.**

**IMPORTANT: PLEASE FILL THIS OUT IMMEDIATELY AFTER COLLECTING YOUR URINE SAMPLE.**

Note: We will NOT test your urine for cannabis or any illegal substances. We only ask about cannabis (and grilled meat) below because they can produce chemicals in your urine similar to smoking and vaping.

DATE OF COLLECTION: _______________ TIME OF COLLECTION: _______________ am / pm

## When was the LAST time you did the following…

*Please circle one response per row. If you have never done the following, select 'Not at all in last 7 days'.*

| | | | | | | |
|---|---|---|---|---|---|---|
| **Used an e-cigarette/vaped** | Less than 1 hour ago | 1-6 hours ago | 7-12 hours ago | 12-24 hours ago | 1-7 days ago | Not at all in last 7 days |
| **Smoked a regular cigarette** | Less than 1 hour ago | 1-6 hours ago | 7-12 hours ago | 12-24 hours ago | 1-7 days ago | Not at all in last 7 days |
| **Smoked any other tobacco** (cigar, cigarillo, bidi, shisha, etc.) | Less than 1 hour ago | 1-6 hours ago | 7-12 hours ago | 12-24 hours ago | 1-7 days ago | Not at all in last 7 days |
| **Smoked cannabis/marijuana** | Less than 1 hour ago | 1-6 hours ago | 7-12 hours ago | 12-24 hours ago | 1-7 days ago | Not at all in last 7 days |
| **Vaped cannabis/marijuana** | Less than 1 hour ago | 1-6 hours ago | 7-12 hours ago | 12-24 hours ago | 1-7 days ago | Not at all in last 7 days |
| **Used smokeless tobacco** (chew, pinch, snuff, snus) | Less than 1 hour ago | 1-6 hours ago | 7-12 hours ago | 12-24 hours ago | 1-7 days ago | Not at all in last 7 days |
| **Used nicotine replacement therapy** (patches, gum, lozenges, etc.) **or nicotine pouches** | Less than 1 hour ago | 1-6 hours ago | 7-12 hours ago | 12-24 hours ago | 1-7 days ago | Not at all in last 7 days |
| **Ate grilled meat** (i.e, cooked over flame or charcoal, or with black grill marks) | Less than 1 hour ago | 1-6 hours ago | 7-12 hours ago | 12-24 hours ago | 1-7 days ago | Not at all in last 7 days |
| **Were in the presence of someone smoking cigarettes or tobacco inside** (home, car, etc.) | Less than 1 hour ago | 1-6 hours ago | 7-12 hours ago | 12-24 hours ago | 1-7 days ago | Not at all in last 7 days |

## Please tell us more about the LAST e-cigarette you used *(if applicable)*:

1. **What brand was the device?** (*please be as specific as possible*) ________________________________________________
2. **What brand was the cartridge, pod or e-liquid?** (*please be as specific as possible*) ________________________________________________
3. **What flavour was the cartridge, pod or e-liquid?** ________________________________________________
4. a) **Did it contain nicotine?** (*circle one*)
   - Yes →
   - No
   - Don't know

   b) **What concentration of nicotine was it?** (*answer in mg/ml OR %*)

   ________ mg/ml **OR** ______% nicotine

   Don't know

   c) **Was it nicotine salt?** (*circle one*)
   - Yes
   - No
   - Don't know

**PLEASE INCLUDE THIS FORM WITH YOUR URINE SAMPLES IN THE ENVELOPE PROVIDED.**
